# Identification of gene fusions from human lung cancer mass spectrometry data

**DOI:** 10.1186/1471-2164-14-S8-S5

**Published:** 2013-12-09

**Authors:** Han Sun, Xiaobin Xing, Jing Li, Fengli Zhou, Yunqin Chen, Ying He, Wei Li, Guangwu Wei, Xiao Chang, Jia Jia, Yixue Li, Lu Xie

**Affiliations:** 1 Key Laboratory of Systems Biology, Shanghai Institutes for Biological Science, Chinese Academy of Sciences, Shanghai, 200031, China; 2 Shanghai Center for Bioinformation Technology, Shanghai Academy of Science and Technology, Shanghai, 201203, China; 3 Genome Biology Unit, European Molecular Biology Laboratory, Heidelberg, 69117, Germany; 4 Department of Respiration, The Third Affiliated Hospital of Sun Yat-sen University, Guangzhou, 510630, China; 5 Department of Pediatrics, Division of Human Genetics, The Center for Applied Genomics, Children's Hospital of Philadelphia, Philadelphia, PA 19104, USA

## Abstract

**Background:**

Tandem mass spectrometry (MS/MS) technology has been applied to identify proteins, as an ultimate approach to confirm the original genome annotation. To be able to identify gene fusion proteins, a special database containing peptides that cross over gene fusion breakpoints is needed.

**Methods:**

It is impractical to construct a database that includes all possible fusion peptides originated from potential breakpoints. Focusing on 6259 reported and predicted gene fusion pairs from ChimerDB 2.0 and Cancer Gene Census, we for the first time created a database CanProFu that comprehensively annotates fusion peptides formed by exon-exon linkage between these pairing genes.

**Results:**

Applying this database to mass spectrometry datasets of 40 human non-small cell lung cancer (NSCLC) samples and 39 normal lung samples with stringent searching criteria, we were able to identify 19 unique fusion peptides characterizing gene fusion events. Among them 11 gene fusion events were only found in NSCLC samples. And also, 4 alternative splicing events were characterized in cancerous or normal lung samples.

**Conclusions:**

The database and workflow in this work can be flexibly applied to other MS/MS based human cancer experiments to detect gene fusions as potential disease biomarkers or drug targets.

## Introduction

Cancers arise as the result of genomic changes that occur in DNA sequences of cells [1]. These changes include single nucleotide variation (SNV), small insertion and deletion (INDEL), structural variation (SV) including deletion, duplication, inversion, translocation etc., and so on. Non-synonymous SNVs which could cause the variation of amino acid in protein have always been the interest of disease related research in genomics studies [2,3]. Recently, some researchers also tried to identify and validate the non-synonymous SNV in the proteomics level from tandem mass spectrometry data [4,5]. Their difficulty rooted in the present situation that the analysis of mass spectrometry data mainly relied on the database searching strategy. If the mutated peptide were not included in the database, they could not be identified. As for more complicated gene structure variations that cause change of protein translation, such as gene fusion, alternative splicing, it is even more difficult to identify and validate from proteomics level.

SVs that may concatenate two different genes to form a new gene and new protein product are named gene fusions. Fusion genes are often oncogenes, such as *BCR:ABL *in chronic myeloid leukaemia (CML), *TMPRSS2:ERG *in prostate cancer, *EML4:ALK *in non-small-cell lung cancer (NSCLC) and so on. Among them the first discovered and most famous fusion gene is the *BCR:ABL. ABL *and *BCR *are normal genes on chr9 and chr22 respectively and *ABL *encodes a tyrosine kinase whose activity is tightly regulated. However, when the translocation occurred between chr9 and chr22, a phosphate group was added to tyrosine. The result of this event is the formation of *BCR:ABL *whose activity was deregulated. *BCR:ABL *and many other fusion genes exert their tumorigenic action mainly through two mechanisms. The first one is that one gene is concatenated to the promoter of the other gene, and the expression of the downstream gene is affected and regulated by the promoter of the upstream gene. The second one is that partial sequence of one gene was concatenated to the other and they altogether give rise to a new protein product. Obviously, the second mechanism should give opportunity to MS/MS to identify those new proteins if only the targeted fusion peptides could be included in the searched database when identifying proteins from mass spectrometry data, just as the discovery of the SNV peptides [4,5].

The technology of identifying novel proteins and eventually explaining or discovering new events on genome annotation is called proteogenomics [6]. Through proteogenomics genome variation events such as new protein coding region, new alternative splicing, frame-shift translation, N-terminal methionine excision, signal peptides etc. can be studied on both levels of proteomics and genomics. This strategy has already been used in many species including human [7], mouse [8], arabidopsis [9] and many bacteria [10] to help improve genome annotation. Recently the technology has been applied in cancer proteomics data [4]. However, it has not been applied to identify cancer fusion proteins yet.

Lung cancer is both one of the most commonly diagnosed cancers worldwide (1.61 million, 12.7% of the total) and one of the most common causes of cancer death (1.38 million, 18.2% of the total) [11]. It can be classified into two main types: small-cell lung carcinoma (SCLC) and non-small-cell lung carcinoma (NSCLC) and NSCLC includes three main subtypes: adenocarcinoma (ADC, about 40% of lung cancers), squamous-cell lung carcinoma (SCC, about 30% of lung cancers), and large-cell lung carcinoma (about 9% of lung cancers). Lung cancer is most commonly caused by long-term exposure to tobacco smoke, in genetics level recurrent somatic SNVs have been identified, including those in *KRAS, LRP1B, NF1 *and so on[12]. Multiple gene fusions, such as *EML4:ALK, TFG:ALK, SLC34AL:ROS, CD74:ROS *have also been characterized by PCR or next generation sequencing (NGS) technology [13,14].

**Figure 1:**
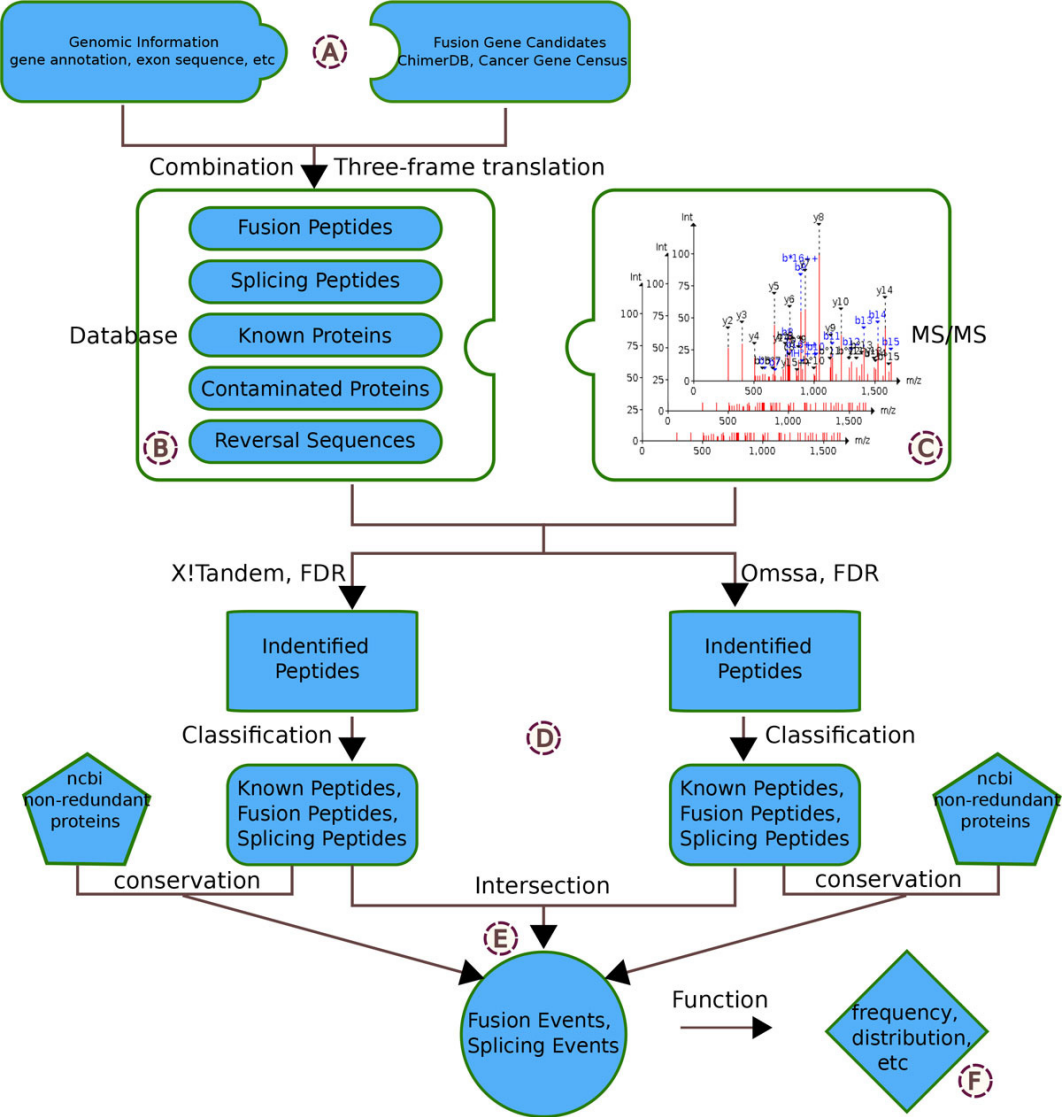
**Workflow for identifying gene fusion and splicing events from MS/MS spectrometry data**. A) The external information used for constructing the searchable database B) Five components of the searchable database C) MS/MS data from 40 non-small cell lung cancer(NSCLC) samples and 39 normal lung samples D) Two search engines and strict false discovery rates (FDR) were used to identify the peptides E) More creditable results from the intersection of two search engines and the conservation information F) The distribution of the identified fusion or splicing events among the caner and normal samples.

To study more thoroughly the possible impact of gene fusions on the genetics of lung cancer, here in this work we used proteogenomics strategy to identify gene fusion proteins based on high-throughput lung cancer proteomics data. To achieve our goal the most important job is to construct a searchable fusion peptide database. From our previous work on construction of searchable peptide database covering all possible splicing events in mouse [8], we knew that it would be impractical to construct a database that includes all possible fusion peptides originated from potential breakpoints. Therefore we utilized the information of fusion gene pairs collected from published papers in Cancer Gene Census[15], and information from database Chimer DB 2.0 [16] which contains some predicted gene pairs from DNA-Seq, RNA-Seq or EST data, and constructed CanProFu --- a specific database for identifying potential gene fusion peptides and alternative splicing peptides based on human mass spectrometry data. Through strict searching strategy, we identified some fusion and splicing events that demonstrate distinctive distribution among ADC, SCC or Normal samples. Our work pipeline is illustrated in Figure 1.

## Results

### CanProFu - the fusion peptide database

From Cancer Gene Census [15] and Chimer DB 2.0 [16], we obtained altogether 6259 non-redundant gene pairs (6174 genes) that can form fusion genes, these are the candidates to construct our potential fusion peptide database --- CanProFu. All the exon sequences, and also the additional information such as exon ID, gene ID, exon position, gene position, gene symbol et al, were obtained from Ensembl Genes 61 using BioMart [17]. We considered only fusion events with break points of both the original pair of genes located in intron regions. The resulted CanProFu is composed of five types of peptides: Fusion (close to five million peptides), Splicing (close to two million peptides), Annotated (about 130 thousand proteins), Contaminated (248 peptides) and Reversal (about 7 million peptides). The total sequences included in CanProFu are about 14 million. Detailed numbers are provided in Additional File 1.

### Fusion peptides and splicing peptides identified in lung cancer based on mass spectrometry data

Mass spectrometry raw data of 20 human lung squamous cell carcinoma, 20 lung adenocarcinoma, and 39 normal lung tissues [31] were downloaded from http://www.ProteomeCommons.org Tranche network [18], and were converted to MGF format by ProteoWizard msconvert tool [19] before submitted to search engines. Two search engines, X!Tandem and Omssa, were applied to the above three types of mass spectrometry data to identify fusion peptides and splicing peptides. After data quality control and cross-reference validation, we consider a peptide as truly reliable only if it passes at least one of the following two criteria: identified by both search engines, or conserved in other species other than homo sapiens (for splicing peptides only). Table 1 shows final 19 fusion peptides and 4 splicing peptides that passed such criteria. The most reliable peptides would be those identified as fully digested with no mis-cleavage, and by both search engines.

**Table 1:** The characterized fusion or splicing peptides identified from the MS/MS data.

	Peptide	Gene	No. X!Tandem	No. Omssa		
Fusion	EQISENPTEATDIDFIR	PTPN12:HSP90AA1	5	9	A	D
Fusion	VIFMDGNGYISAAELR	HN1:CALM1	9	3	A	D
Fusion	IMGIPEEEQMVLSR	MYH9:ALK	4	5	A	D
Fusion	ENVGLEEEQQALQK	PDIA6:TPM2	3	6	A	D
Fusion	AVFVDLEPTVIGGGSVR	TUBA1C:PCGF2	5	3	A	D
Fusion	AAEDDEFTHLYTLIVRPDNTYEVK	GPR115:CALR	2	5	A	D
Fusion	EDSELLISSWLVTDR	DOCK9:BAZ1A	3	2	A	D
Fusion	AVQQELDDLLVDLDHQR	TGFBI:MYH9	2	2	A	D
Fusion	QVTNFLSSINEEITPR	FGFR1:BCR	1	2	A	D
Fusion	ERPAPGQAVLSGGTTMYPGIADR	ACO2:ACTB	1	2	A	D
Fusion	LSAASTWLEDEGVGATTVLFK	HYOU1:HMGA1	1	1	A	D
Fusion	AVFVDLEPTVIEPVR	TUBA1A:PTPN13	1	1	A	D
Fusion	EAREVIELTK	CLCN3:SMNDC1	4	5	B	D
Fusion	NKAEILELAGNAAR	ATP2B4:H2AFY	3	3	B	D
Fusion	EAKGESGPSGPAGPTGAR	USP6:COL1A1	3	2	B	D
Fusion	GRTGDAGPVGEAGAAGPAGPAGPR	COL1A1:COL1A2	1	2	B	D
Fusion	AKQEPEVNGGSGDAVPSG NEVSENMEEEEEALSLMK	NASP:WIPF1	1	2	B	D
Fusion	AHSEEPMEIFVDDETK	ESPN:BAT1	4	6	C	D
Fusion	TTGIVMDSGDGVTHTVPDASRVP	ACTB:GNAS	1	1	C	D
Splicing	INGGGGGSVPGIER	HNRNPM	12	0	A	
Splicing	GDVEEDETIPDSPSVLETIR	TNPO1	2	6	A	D
Splicing	GGSGYGDLGGPIITTQVTIPK	HNRNPK	3	3	A	D
Splicing	RVEDEVNSGVGQDGSLLSSPFLK	SLC35A4	2	2	B	D

The two genes in the fusion events are separated by colon. The value in the columns of No.X!Tandem and No.Omssa column are the number of spectra of the peptide. A indicates that the peptide was fully digested by trypsin and with no mis-cleavage. B indicates that the peptide was fully digested but with one mis-cleavage. C indicates that the peptide was semi-digested. D indicates that the peptide was identified by at least one same spectrum in both X!Tandem and Omssa search engines.

### Data quality control and cross-reference validation of the identified fusion peptides and splicing peptides

False positive discovery is the main obstacle facing identification of peptides from customized database searching. To antagonize this issue in our study we applied data quality control and cross-reference validation. Quality control was achieved by applying two search engines on the same dataset, and setting very stringent local FDR (false discovery rate) control, and other data quality check at the level of amino acids such as number, digestion status and mis-cleavage status. Cross-reference validation was performed in two ways: conservation blast to sequences from other species than homo sapiens(for splicing peptides) and manual check of the original mass spectra.

To reduce false positives and improve the reliability of identified peptides, two popular search engines, X!Tandem [20] and Omssa [21] were used to score each spectrum to the peptides. Other than using normal FDR value such as 0.01, we minimized FDR control to 10^-6 ^in practice, as described in our previous work [8]. Only peptides with at least three amino acids identified along each side of a fusion/splicing point were retained. All the identified peptides were further classified into three types at decreasing reliability: A) full digestion and no mis-cleavage, the most reliable; B) full digestion and one mis-cleavage; C) semi-digestion. As a result, X!Tandem identified 13780 peptides, among them 13503(97.99%) were Annotated, 203(1.47%) were Fusion and 74(0.54%) were Splicing. Omssa identified 8101 peptides, among them 7894(97.44%) were Annotated, 167(2.06%) were Fusion and 40(0.49%) were Splicing. 25 Fusion peptides and 5 Splicing peptides were identified by both X!Tandem and Omssa. We keep these as candidate identified peptides. The venn diagram is displayed in Additional File 2.

Then we performed conservation analysis by blasting all the peptides to NCBI non-redundant database. If a fusion or splicing peptide could be found in other species, even if they were only identified by one of the search engines, they were included as candidate identified peptides. Additional File 3 demonstrates such an example. By this way, no fusion peptide was found to be conservative among other species (which is understandable, fusion peptides are hardly consistent even among one species) (total up still be 25) and one more splicing peptide was saved (total up to 6).

Then we went on to manually check each of the original spectra for these candidate identified fusion and splicing peptides. We observed that sometimes even one same peptide was identified by both search engines, but the original spectra were completely different. These peptides (6 out of 25 Fusion peptides and 2 out of 6 Splicing peptides) may not be fully credible and were ruled out from the following analysis (Additional File 4). Therefore Table 1 shows the final results of 19 fusion peptides and 4 splicing peptides.

**Figure 2:**
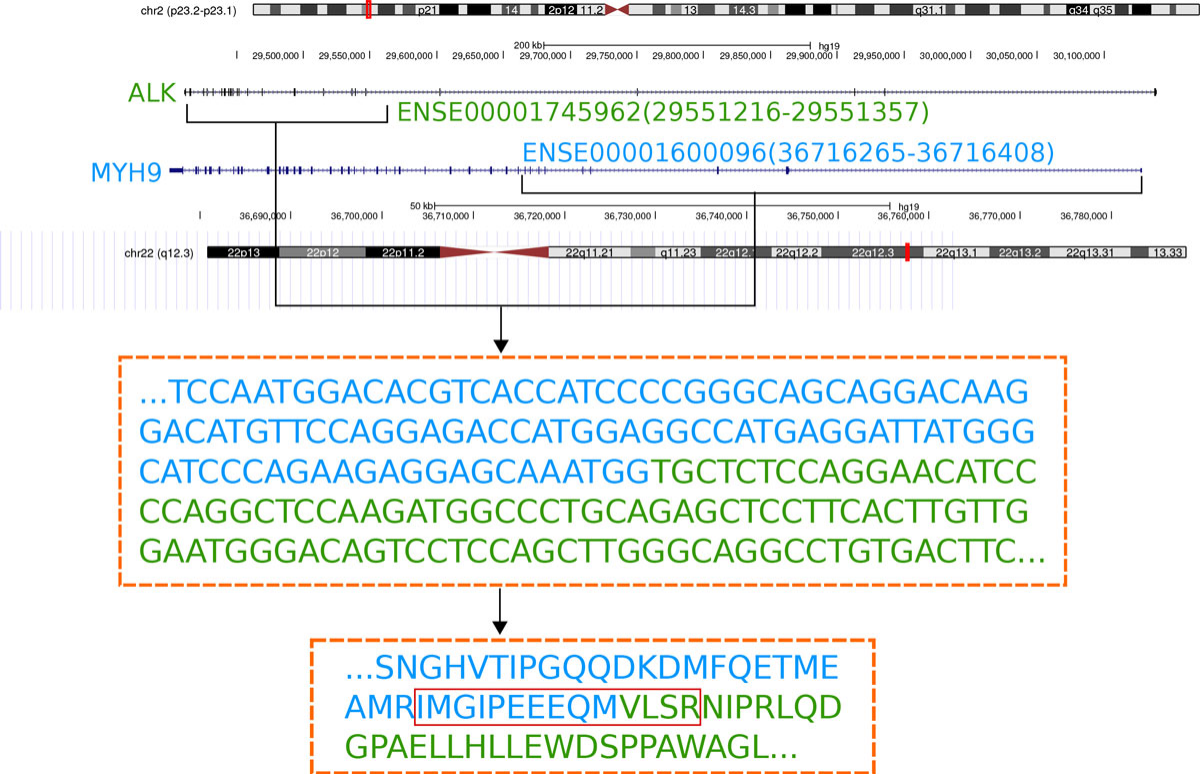
**One example of gene fusion event between MYH9 and ALK**. ENSE00001745962 of ALK is joined with ENSE00001600096 of MYH9. Both the DNA and peptide sequences are shown, with the identified peptide displayed in a red rectangle. The fusion point is separated by different colors.

**Figure 3:**
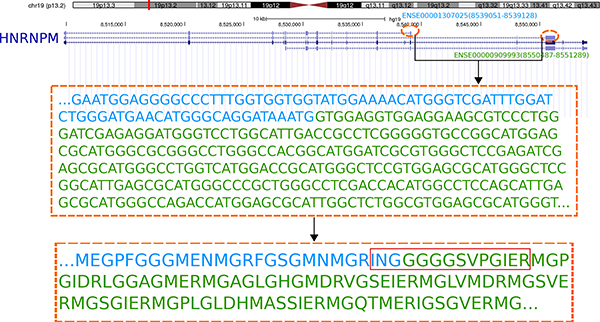
**One example of alternative splicing event in HNRPM**. ENSE00001307025 is joined with ENSE00000909993. Both the DNA and peptide sequences are shown, with the identified peptide displayed in a red rectangle. The splicing point is separated by different colors.

The gene fusion or splicing events could have occurred either in the DNA level or in the RNA level. The original DNA sequences which were translated to identified fusion and splicing peptides could be obtained by backtracking, and the involved exons are shown (Figure 2 and Figure 3).

### The distribution of the fusion peptides and splicing peptides among squamous-cell lung carcinoma (SCC), adenocarcinoma (ADC), and normal lung tissue

**Figure 4:**
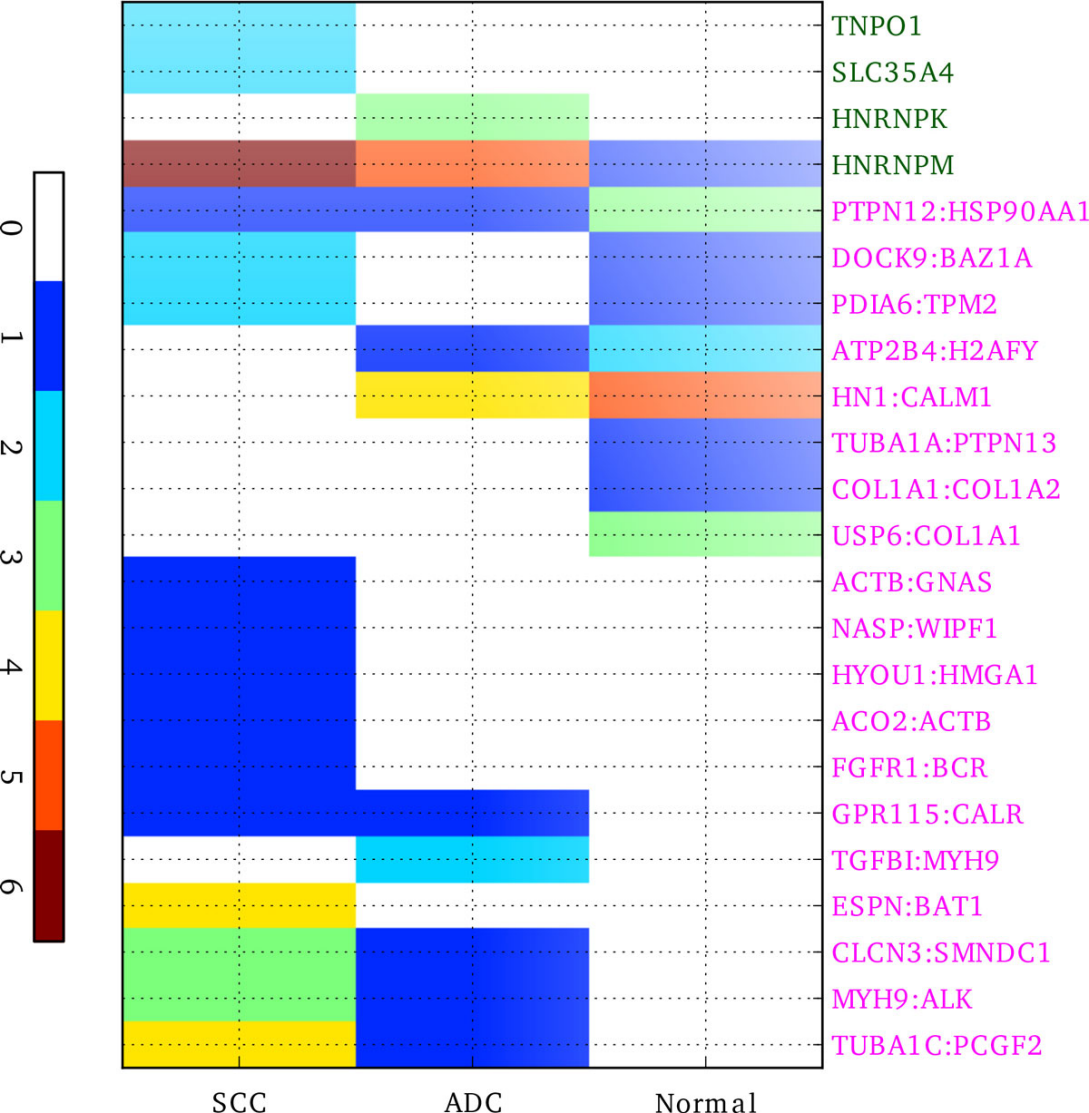
**Distribution of the identified fusion or splicing events among subtypes of NSCLC: SCC (squamous cell carcinoma), ADC (adenocarcinoma), and Normal lung samples**. The two genes in the fusion events are separated by colon and displayed in magenta and the genes related to alternative splicing are displayed in green. The value in the color bar indicates the number of spectrums of the identified peptide.

To determine whether each peptide was from small cell lung carcinoma (SCC), adenocarcinoma (ADC), or normal lung samples (Normal), we went back to check from which type of samples the original mass spectrum came from. Among those 23 peptides (19 of the Fusion peptides and 4 of the Splicing peptides), 8 peptides were only detected in SCC, 2 peptides were only detected in ADC, and 3 peptides were only found in Normal. See Figure 4 and Additional File 5 for detail.

### Functional analyses of *MYH9:ALK *fusion peptide

We found that the peptide which characterizes the fusion event between *MYH9 *and *ALK *genes only existed in SCC and ADC, and higher copies were found in SCC (Figure 4). *MYH9 *normally locates on the complement strand of chr22, and encodes a conventional non-muscle myosin which was reported to be involved in several important functions, such as cytokinesis, cell motility and maintenance of cell shape. Defects in this gene have been associated with non-syndromic sensorineural deafness autosomal dominant type 17 [22], Epstein syndrome and so on [23]. *ALK *normally locates on the complement strand of chr2, and encodes a receptor tyrosine kinase. Many translocations have been found with this *ALK *gene, including *EML4:ALK *which is responsible for approximately 3-5% of non-small-cell lung cancer(NSCLC) [13], *RANBP2:ALK, TPM4:ALK *in inflammatory myofibroblastic tumor [24,25], *NPM1:ALK, ATIC:ALK, TFG:ALK *in anaplastic large cell lymphoma [26-29], and so on. The *MYH9:ALK *which is highlighted in our work was also reported by Lamant et al in anaplastic large cell lymphoma[30], but never in lung cancer before. It may be a novel gene fusion event that plays important role in lung cancer development, and may merit further experimental verification.

## Discussion

In this work, we constructed a database for the purpose of identifying fusion peptides from human cancer proteomics data based on mass spectrometry. We demonstrated the usage of this database by identifying candidate fusion proteins from raw mass spectrometry data generated on human non-small cell lung cancer (NSCLC) subtype samples. Two popular search engines X!Tandem and Omssa were applied. After data quality control and validation by other reference information such as sequences at DNA and RNA level, we eventually identified 19 fusion peptides and 4 splicing peptides, and analyzed their distribution in the original NSCLC subtypes, i.e., squamous cell carcinoma (SCC), adenocarcinoma (ADC), in comparison with that in normal lung sample controls (Normal). *MYH9:ALK *fusion peptide was found to be a novel fusion peptide that occurred in SCC and ADC. The fusion of *MYH9 *and *ALK *gene which resulted in a new protein product might have been an important genomic structural variation that occurred in the development of NSCLC.

Currently genome variation events such as single nucleotide variation (SNV) and structural variation (SV) such like fusion genes are mostly studied by whole genome sequencing (WGS) or RNA sequencing. They have the advantage of being high-throughput. However the disadvantage is that it is uncertain that SNVs or SVs may actually affect disease development by causing final protein product change. To address this, proteomics data must be checked closely. Proteogenomics is such a technique that starts from protein level and traces back to genomic events. It has been tested to help annotate normal genomes across multiple species, by first identifying peptide sequences from mass spectrometry data, and then mapping back to discover the original protein coding events on genome sequences [7,31]. Database searching algorithm is the most often used technique [20,32-34], de novo peptide identification algorithms have also been actively developed [35,36].

Cancer is often referred as a genomic disease, since genomic instability is one major character that leads to malignant cell development. Genomic instability such as single nucleotide mutation, translocation, gene fusion, gene copy number variation etc. causes gene expression change and ultimately changes in proteins. In cancer proteomics field, proteogenomics has been applied to identify SNVs that result in single amino acid variations(SAVs), by constructing specialized database containing SAVs and developing more stringent database searching algorithm [4,5]. However proteogenomics faces substantial challenge when applied to identify more complicated gene structural variations. One gene can have so many translational products, even just by normal alternative splicing [8], not to mention other structural variation such as gene fusions and translocations. To include gene structural variation in any peptide database would increase the search space and false discovery rate, and de novo peptide identification suffers from even lower accuracy. That is why SVs have rarely been studied based on mass spectrometry data.

In this work we presented a primary effort on identifying SV events that resulted in abnormal peptides from cancer mass spectrometry data. To achieve this goal, the first step is to construct a customized database. To make our goal specific and the database at controllable scale, we focused on the purpose of identifying only fusion peptides or splicing peptides. One reason for our experimental design is that gene fusions have been said to occur in all malignancies and account for 20% of human cancer morbidity but all currently reported gene fusions were discovered only through next-generation sequencing in DNA or RNA level or time consuming and small scale experiments in particular proteins[37]. One advantage of the wide study though is that there are multiple resources anchoring gene fusion events that have been studied or predicted in various solid and hematological malignancies, such as ChimerDB [16] and CancergeneCensus [15]. These resources provided us with more reliable materials to construct a cancer fusion peptide database --- CanProFu. We applied more than one database searching algorithm and practiced stringent false discovery rate controlling when testing our database with lung cancer mass spectrometry data.

There are limitations to our work. The samples of subtypes of non-small cell type lung cancer were pooled for mass spectrometry, this made the experimental verification more unfeasible than if the original individual samples were accessible. Our constructed CanProFu is of restricted size and might miss some cancer fusion events that are not included in this primary database. Although we set FDR controlling to be very strict we did not develop special algorithm to antagonize high false discovery rate. All these could be improved in future endeavors, and it may be mentioned that although peptides identified from this work were not experimentally verified because of non-accessibility to original samples, we did identify some splicing peptides from cell line mass spectrometry data and experimentally verified them, which could be a proof of the applicability of our database (data unpublished yet).

Proteogenomics has come a long way in its application of identifying more proteins and explaining more genomic events. Experimentally protein identification coverage has been increased remarkably by industrial development of instruments and improved experimental techniques such as protein digestion by more than one protease [38]. Computationally the construction of more comprehensive database, and attempts to equalize target and decoy database to increase sensitivity when using traditional FDR controlling [39], or modified algorithms of search engines to adapt to different purposes [40] also expanded a great deal the peptide and protein identification rate. Therefore in the future, cancer genomic variations such as gene fusions would be more feasibly identified from proteomics level, based on more extensive mass spectrometry data, expanded customized protein databases, and optimized search engines and algorithms. This would help understand cancer development mechanisms better, and bring cancer biomarker discovery closer to clinical applications.

## Conclusion

For the purpose of identifying cancer fusion events, we constructed a cancer fusion peptide sequence database---CanProFu. Applying mass spectrometry data from 40 non-small cell lung cancer(NSCLC) samples and 39 normal lung tissue controls to search in CanProFu, 19 fusion peptides and 4 splicing peptides were identified. *MYH9:ALK *fusion peptide was newly found and only existed in NSCLC. The CanProFu database and workflow in this work can be flexibly applied to other MS/MS based human cancer experiments to detect gene fusions as potential disease biomarkers and help improve understanding of the related cancer mechanism.

## Methods

### Datasets

Non-small cell lung cancer samples, ADC(adenocarcinoma) and SCC(squamous cell carcinoma) specimens were obtained from pathological Stage I lung cancer patients with no previous cancer history, and Normal specimens were from patients undergoing lung resection for suspicion of lung cancer but not carrying a diagnosis of lung or other cancer. The protein lysates were pooled into four pools: two from non-involved lung tissue (normal control, N = 20 and N = 19, respectively), one from stage I adenocarcinomas (ADC, N = 20), and one from stage I squamous cell carcinomas (SCC, N = 20). Each pool was performed in 4 IEF/RPLC technical replicates. All the fractions were analyzed by LC-MS/MS on an LTQ-Orbitrap hybrid mass spectrometer equipped with an Eksigent 1D Plus NanoLC pump and autosampler. See Kikuchi et al paper for more detailed information [41]. All the RAW data were downloaded from ProteomeCommons.org Tranche network [18], and were converted to MGF format by ProteoWizard msconvert tool [19] before submitted to search engines in our work pipeline.

## Construction of cancer fusion peptide database

### Selecting potential gene pairs

There are N (N >= 20000) protein coding genes in the human genome, even if we only consider situation of paired gene fusion breaking points occurring in both original coding regions, N^2 ^possibilities are not acceptable. Two databases (ChimerDB [16] and CancerGeneCensus [15]) have collected large amount of gene fusion events either reported in cancer research literatures or predicted from EST or next generation sequencing data. The potential gene pairs for fusion in ChimerDB were downloaded from ChimerDB 2.0 and those in CancerGeneCensus were downloaded from COSMIC FTP site (CosmicFusionExport_v58_150312). After filtering out wrong gene symbols and removing redundant information, 6259 unique gene pairs relating to 6174 unique genes were curated as potential fusion peptide forming gene pairs and used to construct CanProFu.

### The concatenating of exons from two genes

To control the scale of the database, at this stage we only considered the situation of fusion breaking points falling into intron regions, since if the break point falls in one exon region, each different location would generate a different translation frame. However intron regions are supposedly to be cut out totally in translation, no matter where the breaking points are as long as they fall into introns (Additional File 6). Based on this rule we constructed our fusion database. Primary tests discovered some of the published fusion peptides such as EML4:ALK [13] and NPM1:ALK [29], and proved that our database is reliable (Additional File 7).

### The necessity of including splicing peptides in the database

Additional File 8 explains why splicing peptides should be included in the database. In the case of two homolog genes (left and right) with similar nucleotide sequences and exons: left gene with exons A, B and C, and right gene with exons A, B and E, obviously, the protein with A and E exons could either be formed by the fusion event of left gene and right gene or just be formed by the splicing event of right gene. If our database contained Fusion only, we would regard this peptide as Fusion event but miss possible Splicing event. Therefore splicing peptides of included genes were added into our database, as competitor components. On the other hand, new splicing events might be found in the database as well, that adds one application to our database.

### Database components

**Figure 5:**
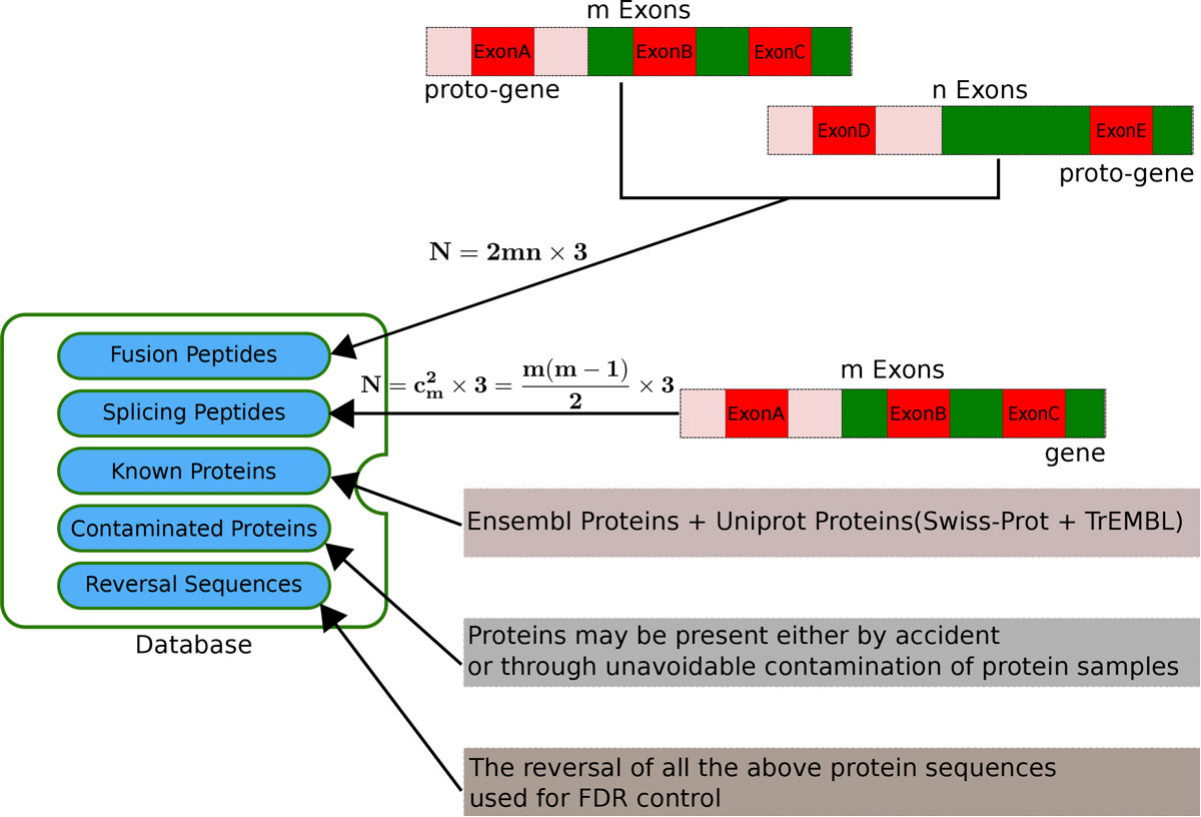
**Five components of the constructed CanProFu database**. Known proteins from both Ensembl and Uniprot and those potential contaminated proteins are used for competition with the main components of the database: Fusion and Splicing. The reversal sequences are used for FDR control.

All the exon sequences, and also the additional information such as exon ID, gene ID, exon position, gene position, gene symbol and so on, of those 6259 gene pairs (6174 genes) were obtained from Ensembl Genes 61 using BioMart [17]. The database contains five parts: Fusion, Splicing, Annotated, Contaminated and Reversal. See Figure 5 for the diagram. The number of the sequences of each component was calculated and provided in Additional File 1. The construction of each component is described briefly here:

### Fusion

a) Each exon sequence of one gene in the gene pairs was concatenated to each exon sequence of the other gene. The upstream and downstream relationship between the two genes of the gene pairs was also considered. The junction point position, exon ID, gene ID, gene symbol and other information were recorded.

b) The concatenated sequences were translated into amino acid sequence using three-frame translation. We did not need to apply six-frame-translation like other works because the direction of exons was already considered.

c) The translated amino acid sequences that don't contain gap (stop condon which is presented as a star in the sequence) before the junction point were retained as fusion peptides. We allow the sequences to have gaps after the junction point, because some fusion events might induce truncation of the translation.

d) The amino acid sequences were tryptic (cutting after K and R, except when either is followed by P) digested into peptides in silico, allowing at most one mis-cleavage at each side of the fusion points.

e) Only the peptides with no less than 6 amino acids crossing fusion points were kept.

### Splicing

The reason why we needed to contain Splicing peptides in the database was explained, and the steps to construct Splicing peptides were basically the same as Fusion except that, in the first step we only concatenated the exon sequences within one gene.

### Annotated

The human protein sequences were downloaded from uniprot (both Swiss-Prot and TrEMBL) [42] and Ensembl.GRCh37.61 [17]. The Annotated protein sequences were used as a competitor component of Fusion and Splicing when searched by search engines.

### Contaminated

Those proteins which may be present either by accident or through unavoidable contamination of samples were also contained as a competitor component. These protein sequences were downloaded from MaxQuant official website [32].

### Reversal

The reversal of all the sequences of the above four parts were constructed for FDR control. The same peptides were merged into one entry, and the peptides were saved in fasta format, with the fusion point position, the gene symbols, the exon ID and other information recorded in the head line of the fasta format file.

## Identification of fusion peptides and splicing peptides

### Database searching and FDR controlling

To reduce false positives and improve the reliability of the results, two popular search engines, X!Tandem [20] and Omssa [21], were used to score the spectrums to the peptides. The default parameters of these search engines were adopted, except that the parent monoisotopic mass error was changed from ± 100da to ± 10da in X!Tandem. Considering that the search space was expanded greatly, and the normal FDR value such as 0.01 may not be suitable, we minimized this value to 10^-6 ^[8]. In practice, for each raw file, the spectrums which were scored to Reversal peptides were counted as F, and the others were counted as T. The results of X!Tandem were sorted by hypescore from high to low and sorted by e-value from low to high for Omssa, then following FDR = 2*F/(T+F), if FDR<=10^-6^, the spectrum matching peptide was considered to have passed the FDR controlling and remained for the following analysis.

### Determination of Fusion, Splicing or Annotated peptides

After FDR controlling, the peptides crossing the Fusion or Splicing points were extracted as candidates. If the candidates belonged to Fusion or Splicing and Annotated simultaneously, they were classified as Annotated, and if the candidates belonged to Fusion and Splicing simultaneously, they were classified as Splicing. Then the Fusion and Splicing peptides were blasted against the NCBI nr database to ensure that they were not known peptides. At the last step, only those peptides which contained at least 3 amino acids at each side of the fusion or splicing point were considered to be able to represent fusion or splicing events and were kept for further analysis.

## Competing interests

The authors declare that they have no competing interests.

## Authors' contributions

Han Sun, Xiaobin Xing and Jing Li carried out the analysis in this study. Han Sun and Lu Xie wrote the manuscript. All authors read and approved the final manuscript.

## Supplementary Material

Additional File 1 **The number of sequences of the five components of our database**. The Annotated part contained known protein sequences from both Uniprot and Ensembl. Click here for file

Additional File 2 **The number of the peptides identified by X!Tandem and Omssa**. Each of the identified peptides could be classified into one of the three types: Annotated which was found in the known proteins, Fusion which crossed over the fusion point of two genes and Splicing which crossed over the alternative splicing point. Click here for file

Additional File 3 **One example of peptide conservation**. The peptide was not in the known human protein, but was found in both Bos Taurus and Desmodus rotundus. This peptide may indicate the alternative splicing event of HNRPM. Click here for file

Additional File 4 **The characterized fusion or splicing peptides identified from the MS/MS data**. The two genes in the fusion events are separated by colon. The value in the columns of No.X!Tandem and No.Omssa column are the number of spectra of the peptide. A indicates that the peptide was fully digested by trypsin and with no mis-cleavage. B indicates that the peptide was fully digested but with one mis-cleavage. C indicates that the peptide was semi-digested. E indicates that the peptide was identified by totally different spectrums in X!Tandem and Omssa search engines. Click here for file

Additional File 5 **Distribution of the identified fusion or splicing events among subtypes of NSCLC: SCC (squamous cell carcinoma), ADC (adenocarcinoma), and Normal lung samples**. The value in the columns of SCC, ADC and Normal column are the number of spectra of the peptide. Click here for file

Additional File 6 **The principle of constructing fusion peptide database: when fusion points fall into intron regions**. The diagram showing both the breakpoints locate in the introns of the two genes. The partial intron sequences (colored in pink and green) between ExonA and ExonE could be removed exactly when translation like the way in the dashed box in lower right corner or couldn't be removed in lower left corner. Click here for file

Additional File 7 **The principle of constructing fusion peptide database: when fusion points fall into intron regions**. Two protein sequences of characterized fusion genes (EML4:ALK and NPM1:ALK) are displayed and the peptides crossing the fusion point do exist in our database where the partial introns were removed completely. Click here for file

Additional File 8 **The diagram indicates why the splicing peptide should also be included in our database**. If the splicing peptides from ExonA and ExonE were not included, then we may regard the identified A/E peptides to be surely from the fusion events. But in fact, they are more likely the result from splicing events. Click here for file

## Declarations

The publication costs for this article were funded by National Key Basic Research Program 2010CB912702.

This article has been published as part of *BMC Genomics *Volume 14 Supplement 8, 2013: Selected articles from the International Conference on Intelligent Biology and Medicine (ICIBM 2013): Genomics. The full contents of the supplement are available online at http://www.biomedcentral.com/bmcgenomics/supplements/14/S8.
