# Supplementary material for: Identification of gene fusions from human lung cancer mass spectrometry data
Source: BMC Genomics. 2013 Dec 9;14(Suppl 8):S5. doi: 10.1186/1471-2164-14-S8-S5 (PMC4042237; doi:10.1186/1471-2164-14-S8-S5)
Supplement: Additional File 1 — The number of sequences of the five components of our database. The Annotated part contained known protein sequences from both Uniprot and Ensembl. [file 1471-2164-14-S8-S5-S1.docx]

**Supplementary Table 1 The number of sequences of the five components of our database.**

| Database | Number of Sequences |
| --- | --- |
| Fusion | 4,930,818 |
| Splicing | 1,897,185 |
| Annotated  (Uniprot + Ensembl) | 122,128  (105,728 + 86,934) |
| Contaminated | 248 |
| Reversal | 6,950,379 |
| Total | 13,900,758 |
