# Supplementary figures and images for: Identification of gene fusions from human lung cancer mass spectrometry data
Source: BMC Genomics. 2013 Dec 9;14(Suppl 8):S5. doi: 10.1186/1471-2164-14-S8-S5 (PMC4042237; doi:10.1186/1471-2164-14-S8-S5)

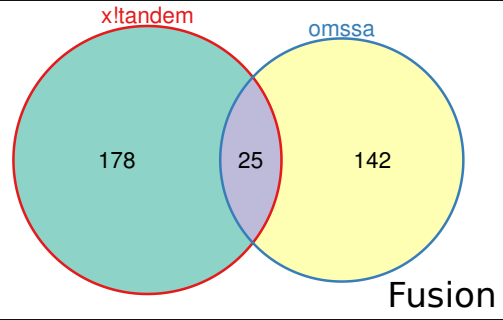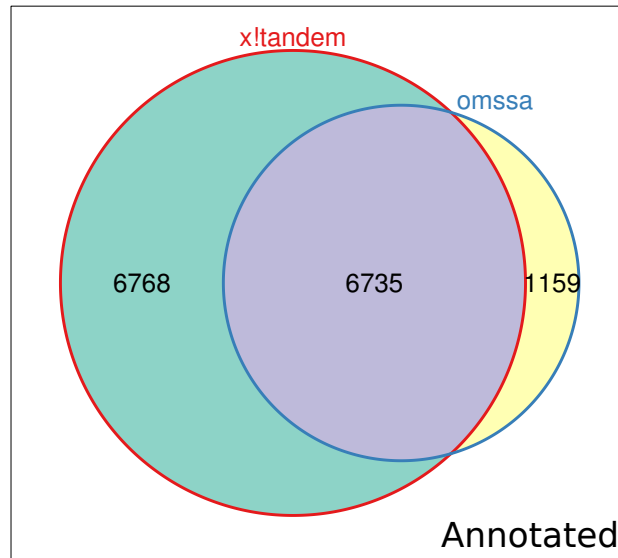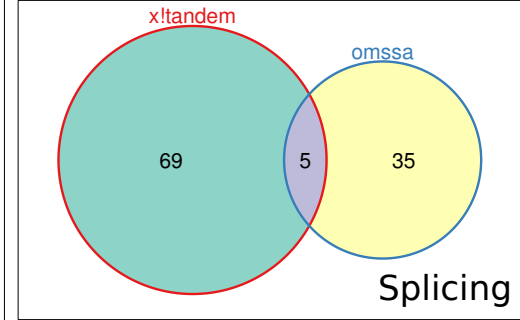

Supplement: Additional File 2 — The number of the peptides identified by X!Tandem and Omssa. Each of the identified peptides could be classified into one of the three types: Annotated which was found in the known proteins, Fusion which crossed over the fusion point of two genes and Splicing which crossed over the alternative splicing point. [file 1471-2164-14-S8-S5-S2.PDF]

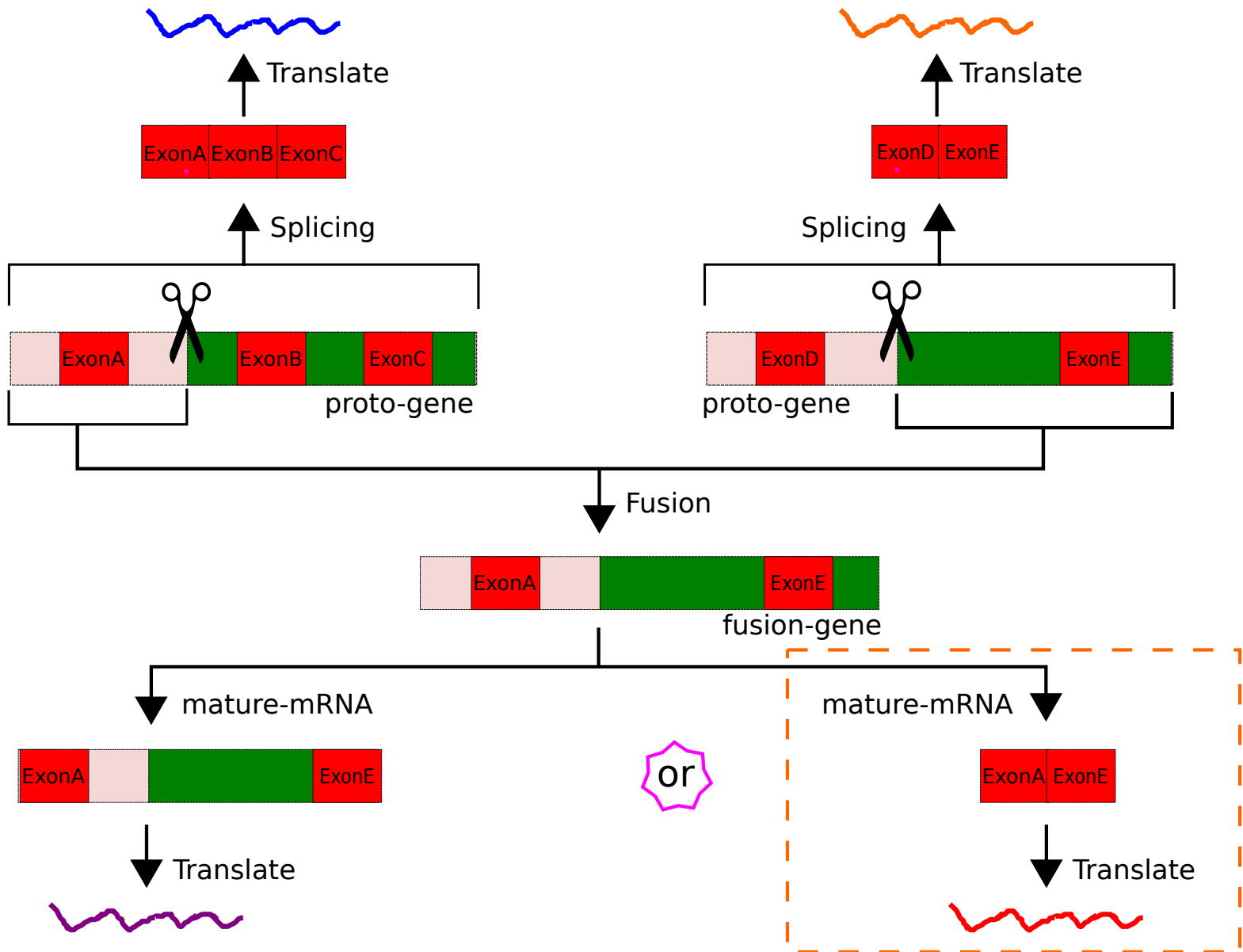

Supplement: Additional File 6 — The principle of constructing fusion peptide database: when fusion points fall into intron regions. The diagram showing both the breakpoints locate in the introns of the two genes. The partial intron sequences (colored in pink and green) between ExonA and ExonE could be removed exactly when translation like the way in the dashed box in lower right corner or couldn't be removed in lower left corner. [file 1471-2164-14-S8-S5-S6.PDF]

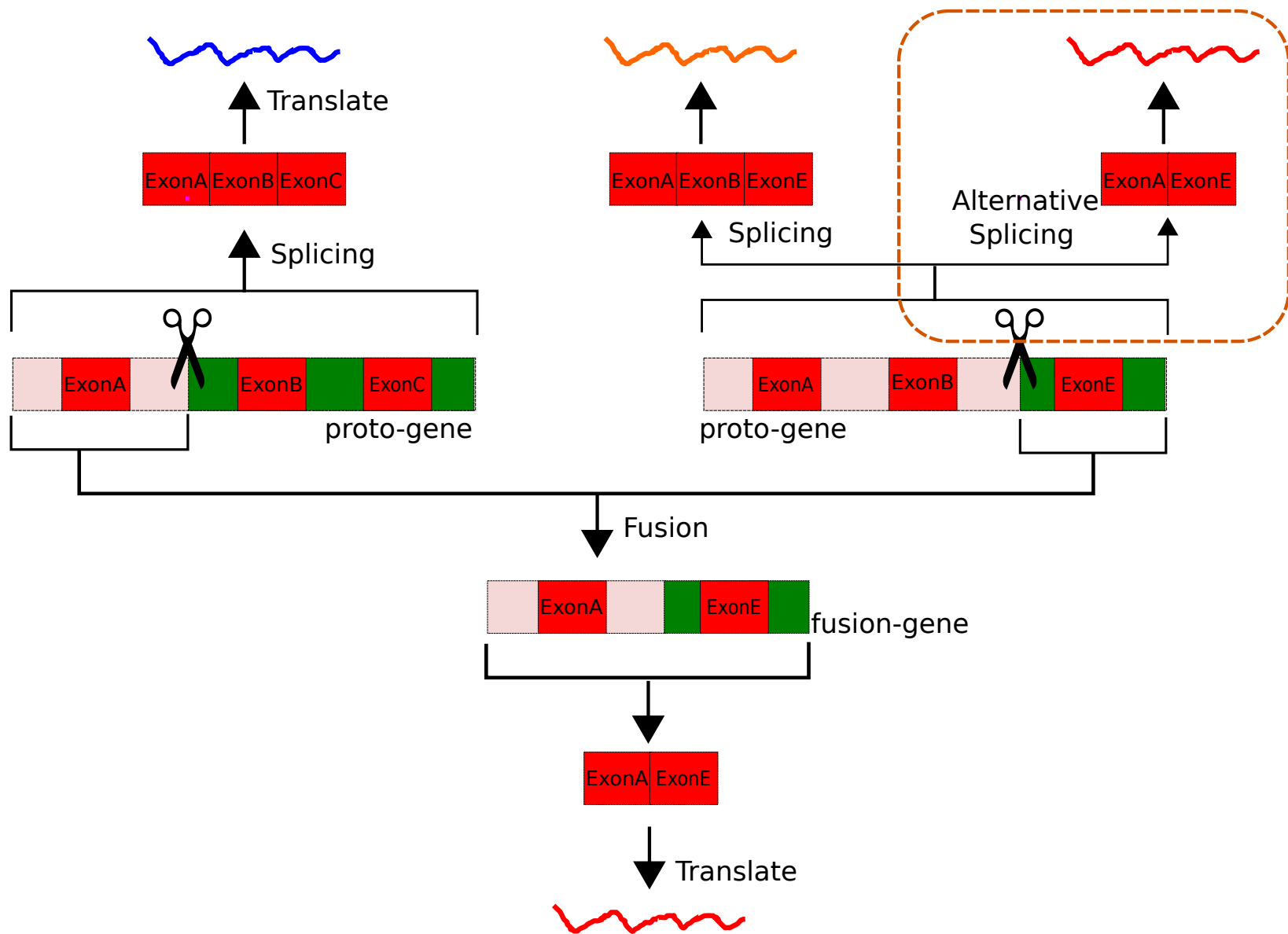

Supplement: Additional File 8 — The diagram indicates why the splicing peptide should also be included in our database. If the splicing peptides from ExonA and ExonE were not included, then we may regard the identified A/E peptides to be surely from the fusion events. But in fact, they are more likely the result from splicing events. [file 1471-2164-14-S8-S5-S8.PDF]
