# Supplementary material for: Identification of gene fusions from human lung cancer mass spectrometry data
Source: BMC Genomics. 2013 Dec 9;14(Suppl 8):S5. doi: 10.1186/1471-2164-14-S8-S5 (PMC4042237; doi:10.1186/1471-2164-14-S8-S5)
Supplement: Additional File 3 — One example of peptide conservation. The peptide was not in the known human protein, but was found in both Bos Taurus and Desmodus rotundus. This peptide may indicate the alternative splicing event of HNRPM. [file 1471-2164-14-S8-S5-S3.PDF]

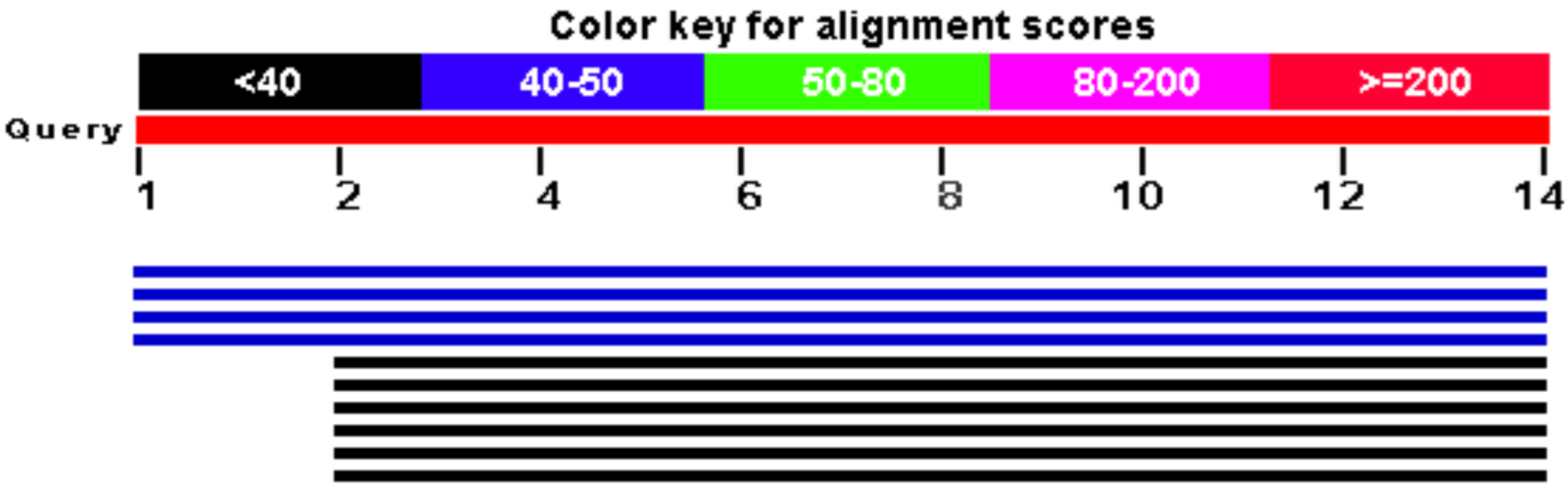

HNRPM protein [Bos taurus]	44.3	62.8	100%	3e-04	100%	AAI03345.2
TPA: heterogeneous nuclear ribonucleoprotein M isoform 1 [Bos taurus]	44.3	62.8	100%	3e-04	100%	DAA27878.1
Putative rna-binding protein hnrnp-m, partial [Desmodus rotundus]	44.3	62.8	100%	3e-04	100%	JAA52537.1
Putative rna-binding protein hnrnp-m [Desmodus rotundus]	44.3	62.8	100%	3e-04	100%	JAA48823.1
unnamed protein product [Homo sapiens]	37.5	37.5	85%	0.043	100%	BAG57075.1
PREDICTED: heterogeneous nuclear ribonucleoprotein M-like, partial [Ornithorhynchus anatinus]	37.5	56.0	85%	0.044	100%	XP_001516207.2

Peptide: INGGGGGSVPGIER

Conservation: Bos taurus(100%), Desmodus rotundus(100%), Homo sapiens(85%)
