# Supplementary material for: Identification of gene fusions from human lung cancer mass spectrometry data
Source: BMC Genomics. 2013 Dec 9;14(Suppl 8):S5. doi: 10.1186/1471-2164-14-S8-S5 (PMC4042237; doi:10.1186/1471-2164-14-S8-S5)
Supplement: Additional File 4 — The characterized fusion or splicing peptides identified from the MS/MS data. The two genes in the fusion events are separated by colon. The value in the columns of No.X!Tandem and No.Omssa column are the number of spectra of the peptide. A indicates that the peptide was fully digested by trypsin and with no mis-cleavage. B indicates that the peptide was fully digested but with one mis-cleavage. C indicates that the peptide was semi-digested. E indicates that the peptide was identified by totally different spectrums in X!Tandem and Omssa search engines. [file 1471-2164-14-S8-S5-S4.docx]

**Supplementary Table 2 The characterized fusion or splicing peptides identified from the MS/MS data.** The two genes in the fusion events are separated by colon. The value in the columns of No.X!Tandem and No.Omssa column are the number of spectra of the peptide. A indicates that the peptide was fully digested by trypsin and with no mis-cleavage. B indicates that the peptide was fully digested but with one mis-cleavage. C indicates that the peptide was semi-digested. E indicates that the peptide was identified by totally different spectrums in X!Tandem and Omssa search engines.

|  | Peptide | Gene | No.  X!Tandem | No. Omssa |  |  |
| --- | --- | --- | --- | --- | --- | --- |
| Fusion | DGEAGAQGPPGPAGLQER | COL1A1:EMILIN1 | 98 | 5 | A | E |
| Fusion | AGVMGSPGEQGPSGASGPAGPR | COL1A2:COL1A1 | 5 | 2 | A | E |
| Fusion | GFSGLQGPPGPPGEPGVV  GAVGTAGPSGPSGLPGER | COL1A1:COL1A2 | 3 | 1 | A | E |
| Fusion | GEEGPSGPVGPAGAVGPR | MYO1C:COL1A2 | 3 | 1 | A | E |
| Fusion | LAEMPAADQEAGGQR | ATP6V1A:PTRF | 2 | 1 | A | E |
| Fusion | AAAAAALQALK | C9orf30:CHMP4B | 1 | 1 | A | E |
| Splicing | GFPGGSPGEQGPSGASGPAGPR | COL1A1 | 1 | 1 | A | E |
| Splicing | RVEDEVNSGVGQDGSLLSSPFLK | SLC35A4 | 2 | 2 | B | E |
