# Supplementary material for: Identification of gene fusions from human lung cancer mass spectrometry data
Source: BMC Genomics. 2013 Dec 9;14(Suppl 8):S5. doi: 10.1186/1471-2164-14-S8-S5 (PMC4042237; doi:10.1186/1471-2164-14-S8-S5)
Supplement: Additional File 5 — Distribution of the identified fusion or splicing events among subtypes of NSCLC: SCC (squamous cell carcinoma), ADC (adenocarcinoma), and Normal lung samples. The value in the columns of SCC, ADC and Normal column are the number of spectra of the peptide. [file 1471-2164-14-S8-S5-S5.docx]

**Supplementary Table 3** **Distribution of the identified fusion or splicing events among subtypes of NSCLC: SCC (squamous cell carcinoma), ADC (adenocarcinoma), and Normal lung samples.** The value in the columns of SCC, ADC and Normal column are the number of spectra of the peptide.

|  | Gene | SCC | ADC | Normal |
| --- | --- | --- | --- | --- |
| Fusion | TUBA1C:PCGF2 | 4 | 1 | 0 |
| Fusion | MYH9:ALK | 3 | 1 | 0 |
| Fusion | CLCN3:SMNDC1 | 3 | 1 | 0 |
| Fusion | ESPN:BAT1 | 4 | 0 | 0 |
| Fusion | TGFBI:MYH9 | 0 | 2 | 0 |
| Fusion | GPR115:CALR | 1 | 1 | 0 |
| Fusion | FGFR1:BCR | 1 | 0 | 0 |
| Fusion | ACO2:ACTB | 1 | 0 | 0 |
| Fusion | HYOU1:HMGA1 | 1 | 0 | 0 |
| Fusion | NASP:WIPF1 | 1 | 0 | 0 |
| Fusion | ACTB:GNAS | 1 | 0 | 0 |
| Fusion | USP6:COL1A1 | 0 | 0 | 3 |
| Fusion | COL1A1:COL1A2 | 0 | 0 | 1 |
| Fusion | TUBA1A:PTPN13 | 0 | 0 | 1 |
| Fusion | HN1:CALM1 | 0 | 4 | 5 |
| Fusion | ATP2B4:H2AFY | 0 | 1 | 2 |
| Fusion | PDIA6:TPM2 | 2 | 0 | 1 |
| Fusion | DOCK9:BAZ1A | 2 | 0 | 1 |
| Fusion | PTPN12:HSP90AA1 | 1 | 1 | 3 |
| Splicing | HNRNPM | 6 | 5 | 1 |
| Splicing | HNRNPK | 0 | 3 | 0 |
| Splicing | SLC35A4 | 2 | 0 | 0 |
| Splicing | TNPO1 | 2 | 0 | 0 |
