# Supplementary material for: Identification of gene fusions from human lung cancer mass spectrometry data
Source: BMC Genomics. 2013 Dec 9;14(Suppl 8):S5. doi: 10.1186/1471-2164-14-S8-S5 (PMC4042237; doi:10.1186/1471-2164-14-S8-S5)
Supplement: Additional File 7 — The principle of constructing fusion peptide database: when fusion points fall into intron regions. Two protein sequences of characterized fusion genes (EML4:ALK and NPM1:ALK) are displayed and the peptides crossing the fusion point do exist in our database where the partial introns were removed completely. [file 1471-2164-14-S8-S5-S7.PDF]

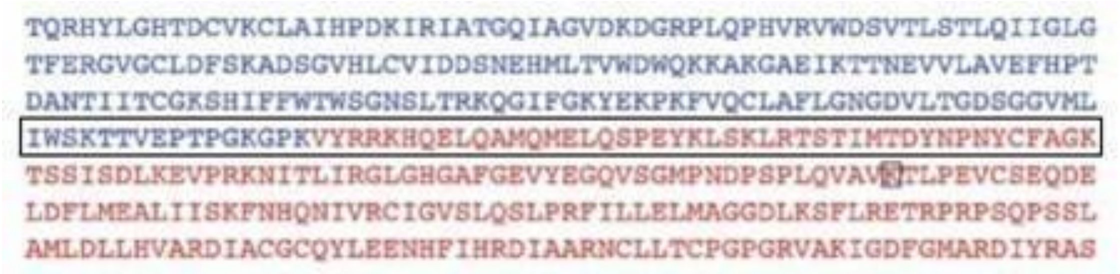


```
>genefusions:ALK:EML4:ENSE00001735043:ENSE00000962700:53:136|2|17|0|45|
LHQ*PEGGAAEKHHPHSKYEKPKFVQCLAFLGNGDVLTGDSGGVMLIWSKTTVEPTPGKGPK
>genefusions:EML4:ALK:ENSE00000962700:ENSE00001154407:136:187|0|46|-2|61|
KYEKPKFVQCLAFLGNGDVLTGDSGGVMLIWSKTTVEPTPGKGPKVYRRKHQELQAMQMELQSPEYKLSKLRTSTIMTDYNPNYCFAGKT
>genefusions:EML4:ALK:ENSE00000962700:ENSE00001154407:136:187|1|45|0|62|
NMKSQNLCSV*HSWGMEMFLLETQVESCLYGAKLL*SPHLGKDLKCTAGSTRSCKPCRWSCRALSTS*ASSAPRPS*PTTTPTTALLARP
```

EML4:ALK, Soda et al, Nature, 2007

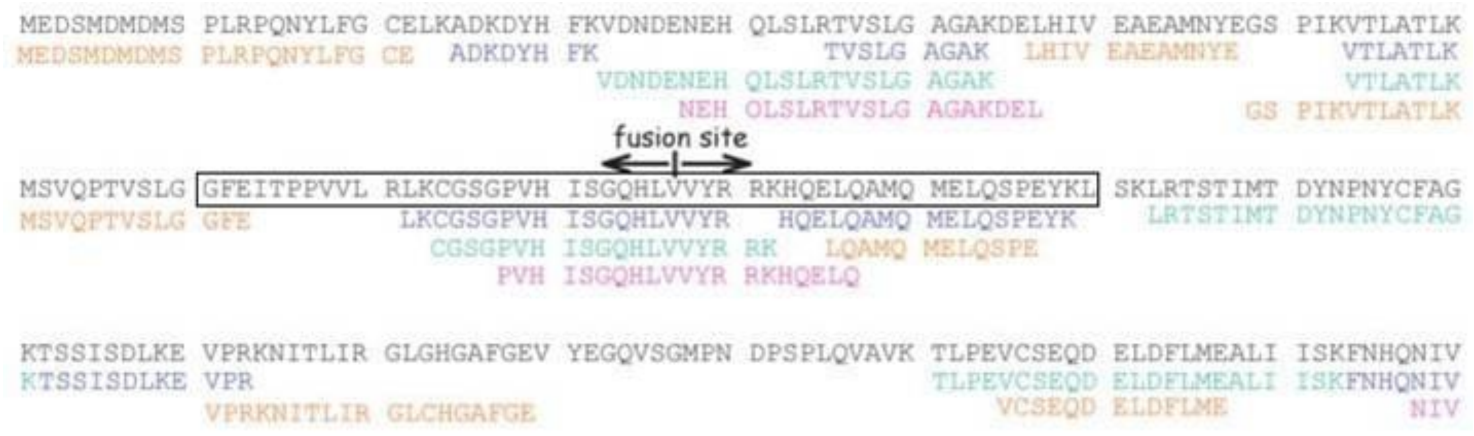


```
>genefusions:ALK:NPM1:ENSE00001735043:ENSE00001084440:53:94|2|17|0|31|
LHQ*PEGGAAEKHHPHSVSLGGFEITPPVVLRLKCGSGPVHISGQHLV
>genefusions:NPM1:ALK:ENSE00001084440:ENSE00001154407:94:187|0|32|-2|61|
VSLGGFEITPPVVLRLKCGSGPVHISGQHLVVYRRKHQELQAMQMELQSPEYKLSKLRTSTIMTDYNPNYCFAGKTSSISDLKEVPRKNITLI
>genefusions:NPM1:ALK:ENSE00001084440:ENSE00001154407:94:187|1|31|0|62|
FPLGALK*HHQWS*G*SVVQGQCILVDST**CTAGSTRSCKPCRWSCRALSTS*ASSAPRPS*PTTTPTTALLARPPPSVT*RRCRGKTSPSF
```

NPM1:ALK, Elenitoba-Johnson et al, PNAS, 2006
